# A novel SGRT system for real‐time optical surface tracking or guidance

**DOI:** 10.1002/acm2.70138

**Published:** 2025-07-13

**Authors:** Xinmin Liu, Daniel Alexander, Daniel Kayser, Thomas Speck, Rodney D. Wiersma

**Affiliations:** ^1^ Department of Radiation Oncology UCLA Los Angeles California USA; ^2^ Department of Radiation Oncology University of Pennsylvania Philadelphia USA; ^3^ LAP GmbH Laser Applikationen Lüneburg Germany

**Keywords:** robustness, SGRT, surface tracking, tracking

## Abstract

**Purpose:**

Optical 3D surface imaging is a technique that reconstructs a patient's surface without requiring external markers. This study aimed to evaluate the static and dynamic accuracy, as well as performance under varying conditions, including changes in skin surface color, ambient room lighting, and the size of the tracked region of interest (ROI), for a novel surface guided radiation therapy (SGRT) system (LUNA 3D).

**Methods:**

The LUNA 3D consisted of three ceiling mounted camera pods, with each pod equipped with a pattern projector and two camera units. Blue 465 nm light projection, combined with narrow bandpass filters and advanced triggering, were used to improve tracking accuracy to surface color and ambient room light variations. GPU processing enabled surface acquisition at high speeds. To investigate static and dynamic accuracy, a 6‐degree‐of‐freedom (6DoF) robot stage was used to move head phantoms to known positions in 6D space. A 6D infrared (IR) tracking camera was employed as a secondary verification tool.

**Results:**

When tracking a static head phantom with a clinically relevant ROI, the LUNA 3D demonstrated a translational noise root‐mean‐sqaured‐error (RMSE) of approximately 0.04 mm LAT, 0.06 mm LNG, and 0.04 mm VRT and a rotational noise RMSE of 0.04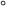 pitch, 0.04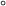 roll, and 0.04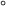 yaw. For varying surface colors, ambient light conditions, and ROI sizes, LUNA 3D's performance, when compared to the IR camera, exhibited a maximum difference of 0.14 mm. The measured frame rate remained approximately constant at 12.5 fps for all ROI sizes tested.

**Conclusion:**

The LUNA 3D demonstrated high sub‐millimeter and sub‐degree tracking accuracy, comparable to or better than that of an IR tracking camera, while exhibiting minimal sensitivity to surface color and ambient lighting conditions. Additionally, it maintained stable and consistent frame rate tracking regardless of the tracked ROI size.

## 1 INTRODUCTION

Optical surface guidance can play a pivotal role in patient positioning for radiation therapy by providing precise and noninvasive methods to ensure accurate alignment and reproducibility during treatment.^1^, ^2^, ^3^ Unlike traditional methods reliant on tattoo marks or manual measurements, 3D surface imaging systems generate detailed representations of a patient's external anatomy while maintaining similar or improved accuracy and decreasing setup time.^4^, ^5^ These images allow for real‐time monitoring and assessment of patient positioning, enabling radiation therapists to verify the exact positioning required for treatment delivery. By comparing these images to a reference 3D surface, deviations in patient setup can be detected and corrected. This minimizes the risk of setup errors and ensures that radiation beams target the intended volume while minimizing exposure to healthy tissues.^6^ 3D surface imaging can be used for daily initial positioning, intrafractional motion monitoring, and radiation beam gating.^7^, ^8^, ^9^

Various methods of optical 3D surface imaging include laser scanning, time‐of‐flight, stereovision, and structured light imaging.^10^, ^11^, ^12^, ^13^ In surface‐guided radiation therapy (SGRT), two predominant methods are employed: pseudo‐random speckled light patterns using stereophotogrammetry and structured light patterns projected onto the patient's skin. Stereovision, employing dual cameras with an unknown speckled light pattern, aids triangulation, while a known structured light pattern can be reconstructed using a single camera. Typically, multiple camera “pods” are integrated into the RT treatment room to widen the field‐of‐view, minimize camera gantry occlusion, and to capture additional surface features in order to enhance registration accuracy. Registration algorithms, categorized as either rigid or deformable, are used to align the patient's surface with a reference surface in six degrees‐of‐freedom (6DoF), enabling determination of translational and rotational shifts. Align surfaces; rigid algorithms concentrate on user‐defined regions of interest (ROIs), while deformable algorithms use the entire surface area, emphasizing the isocenter's position and incorporating depth correction.

The performance of SGRT systems have been found to degrade with changes in surface color and ambient room lighting.^1^, ^14^, ^15^, ^16^ A typical issue is accurate rendering of the 3D surface for a wide range of skin tones encountered in the clinic. For very dark skin tones, the surface rendered will often have missing regions that cannot be reconstructed due to camera hardware limitations in that the reflected light signal can no longer be amplified enough to be detected (i.e., the exposure time limit has been reached). Various strategies have been employed to correct this issue, which include: manually adjusting camera apertures to minimize this effect, a software option where end users select the skin type (light, dark) that most closely matches the patient, and combining thermal imaging with surface imaging in order to stabilize measurements.^1^, ^10^ Other limitations with current SGRT systems also include limited 3D surface size and slow or varying frame rates.^17^, ^18^

In this work, we investigate a novel SGRT system, LUNA 3D, which has been recently developed. It utilizes blue light projection, narrow band‐pass filters, and advanced triggering to enable precise position tracking across a variety of surface colors and ambient lighting conditions. The tracking accuracy of the LUNA 3D system was evaluated using two head phantoms positioned at various orientations on a 6DoF robotic platform within a predefined space. A 6D infrared (IR) tracking camera was simultaneously employed as a secondary position verification system. The purpose of this study was to assess the static and dynamic accuracy of the LUNA 3D system, along with its performance under varying conditions, including changes in skin surface color, ambient room lighting, and the size of the tracked ROI.

## 2 MATERIALS AND METHODS

The LUNA 3D (LAP GmbH, Germany) SGRT system consisted of three camera pods, with each pod containing a speckle pattern projector and two camera units. The use of multiple pods allows for compatibility with various patient surface geometries and gantry positions, allowing for a given ROI to be visible under various treatment conditions. An image resolution of 5 megapixels (2448 × 2048 CMOS array with a 3.45 μm pixel size) for each camera unit was found to provide adequate surface imaging quality. In order to improve the data quality for different surface colors and improve robustness to variations in ambient lighting, the system incorporated a blue projection wavelength (465 nm), narrow bandpass filters, and synchronized triggering. The use of blue light projection ensures high visibility, even on dark surfaces, making it particularly suitable for applications involving diverse skin tones or materials with low reflectivity. On human skin, blue light has a relatively shallow penetration depth due to its shorter wavelength compared to other colors.^19^ This characteristic enhances the resolution of surface projection, producing sharp and bright projections critical for precise imaging. Narrow bandpass filters, finely tuned to the blue light wavelength, were employed to eliminate interference from background illumination, ensuring consistent performance regardless of room lighting conditions. Furthermore, these filters enhance contrast by isolating the projection's wavelength, improving the accuracy of subsequent image processing. Advanced triggering mechanisms for camera pods were synchronized with the pod projectors to prevent any overlap of projection patterns from multiple pods. Such overlaps could otherwise degrade image quality by introducing artifacts or blurring, compromising the sharpness and accuracy of structural details.

**FIGURE 1:**
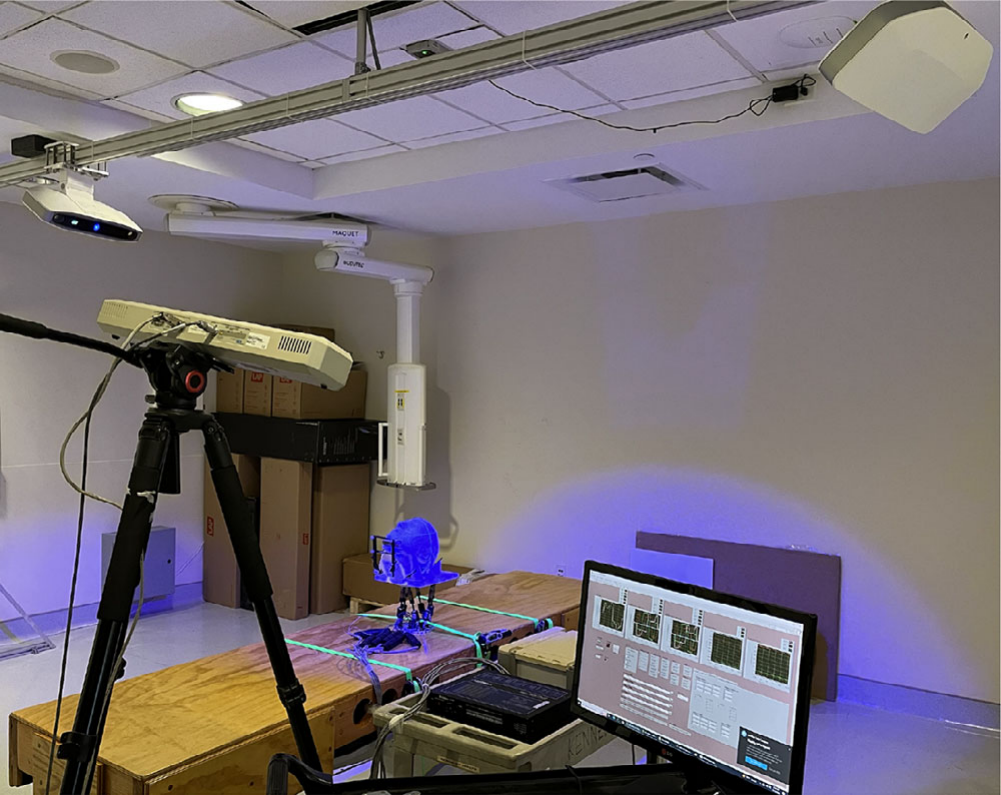
Mock Linac RT setup environment with a three camera pod LUNA 3D surface tracking system, IR camera, high accuracy 6D robot, and white color head phantom. IR, infrared; Linac, linear accelerator.

Up to four camera pods can be used with the LUNA 3D in order to maximize viewable surface area. For a C‐Arm linear accelerator (Linac) a three pod arrangement is used with one pod mounted centrally at the foot side, and two pods mounted laterally. For bore based Linacs, a four pod arrangement is used where two pods are mounted centrally at the foot and head side to view down the bore, and the other two pods mounted laterally. For this work a mock RT treatment room environment was created assuming a C‐Arm Linac setup with the three pod arrangement with lateral pods angled 85 degrees with respect to the foot pod (Figure 1). All camera pods were focused on a mock Linac isocenter as defined by wall mounted positioning lasers. To calibrate the system, each camera pod was first synchronized through image acquisition with the speckle pattern projection. After this all camera pods were synchronized together by capturing a set of six 2D images of a cubic phantom (EasyCube, LAP GmbH, Germany) oriented on top of a 2D pattern board.

Surface reconstruction was done by processing the full 2D image set of all camera units of a scene and identifying corresponding points to create a 3D surface. With up to four camera pods (eight cameras) running simultaneously at 12.5Hz frames per second (fps), the maximum data transfer rate can be greater than 3 Gbit/s. In order to process and analyze the input data in real‐time, a GPU (Nvidia, CA) with 16384 cores, 2.23 GHz, and 24 GB memory was used. This allowed a joint 3D surface mesh at a frame rate of 12.5 Hz to be generated. A web browser‐based graphical user interface (GUI) was provided through a workstation backend allowing multiple web browser compatible front‐end devices (desktops, tables, etc.) for visualization and user interaction. In the mock RT room a typical system configuration was used with a control room desktop, a setup monitor in the treatment room, a tablet device for user interaction in the treatment room. The measured live 3D surface data displayed on the LUNA 3D GUI was compared to a reference surface data using a rigid iterative closest point (ICP) registration algorithm to calculate the 6D transformation matrix between the two surfaces. Reference surfaces could either be captured by the LUNA 3D system or importing from preexisting DICOM RT data. For DICOM data the external contour from the structure set and isocenter information from the RT plan can be used to generate the reference surface. In this study, all ROI reference surfaces were directly captured using the LUNA 3D device.

### 2.1 Spatial tracking performance

To characterize spatial tracking performance, two different colored (black and white) head phantoms were used and moved to well defined positions in 6D space while undergoing real‐time tracking by the LUNA 3D system (Figure 1). A high accuracy 6DoF Stewart platform (hexapod) robot was built to perform highly accurate motion using inverse kinematics control.^20^, ^21^, ^22^ The accuracy of the robot was previously verified to have a translational root mean square error (RMSE) of 0.14, 0.22, and 0.08 mm for LAT, LNG, and VRT, respectively, and a rotational RMSE of 0.16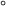, 0.06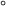, and 0.08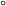 for pitch, roll, and yaw, respectively.^20^ The robotic stage was employed to move the head phantom within a translational space measuring 20×20×20 ${\mathrm{mm}}^{3}$, centered at the Linac iso‐center. This evaluation space was evenly divided into a grid, consisting of 3 points along each lateral (LAT) and vertical (VRT) axis, and 4 points along the longitudinal (LNG) axis. Evaluation started from the iso‐center and came back at the same point, totaling 38 grid points. At each grid point, the robot stopped and held the head phantom stationary for 8s while the camera reported 6D position was sampled. Similarly, for rotational accuracy evaluation, the robot moved the head phantoms to 38 gridded points within a rotational space of 16×16×16 ${\text{degrees}}^{3}$. In certain tests involving various clinical conditions, a cube motion for the head phantoms was used. In these scenarios, the robot moved from the LINAC iso‐center to each of the eight vertices of a 20mm‐side cube and then returned to the iso‐center. In addition, to test camera tracking noise and positional drift over time, the robot was used to hold the phantom in a static position at Linac isocenter.

As a secondary phantom position verification system, the head phantom was simultaneously tracked in 6D using by an IR camera (Polaris, Northern Digital Incorporated, Canada).^23^ Based on manufacture specifications, the IR marker tracking system has a tracking accuracy of less than 0.25 mm RMSE for both passive and active markers located up to 2.4 m from the camera system. The fps of the IR system is around 12 Hz which was communicated over RS‐232 bus to the control computer. As shown in Figure 1, IR markers were rigidly fixed to the bottom of the phantom to avoid obscuring the surface view of the LUNA 3D.

### 2.2 Varying clinical conditions

**FIGURE 2:**
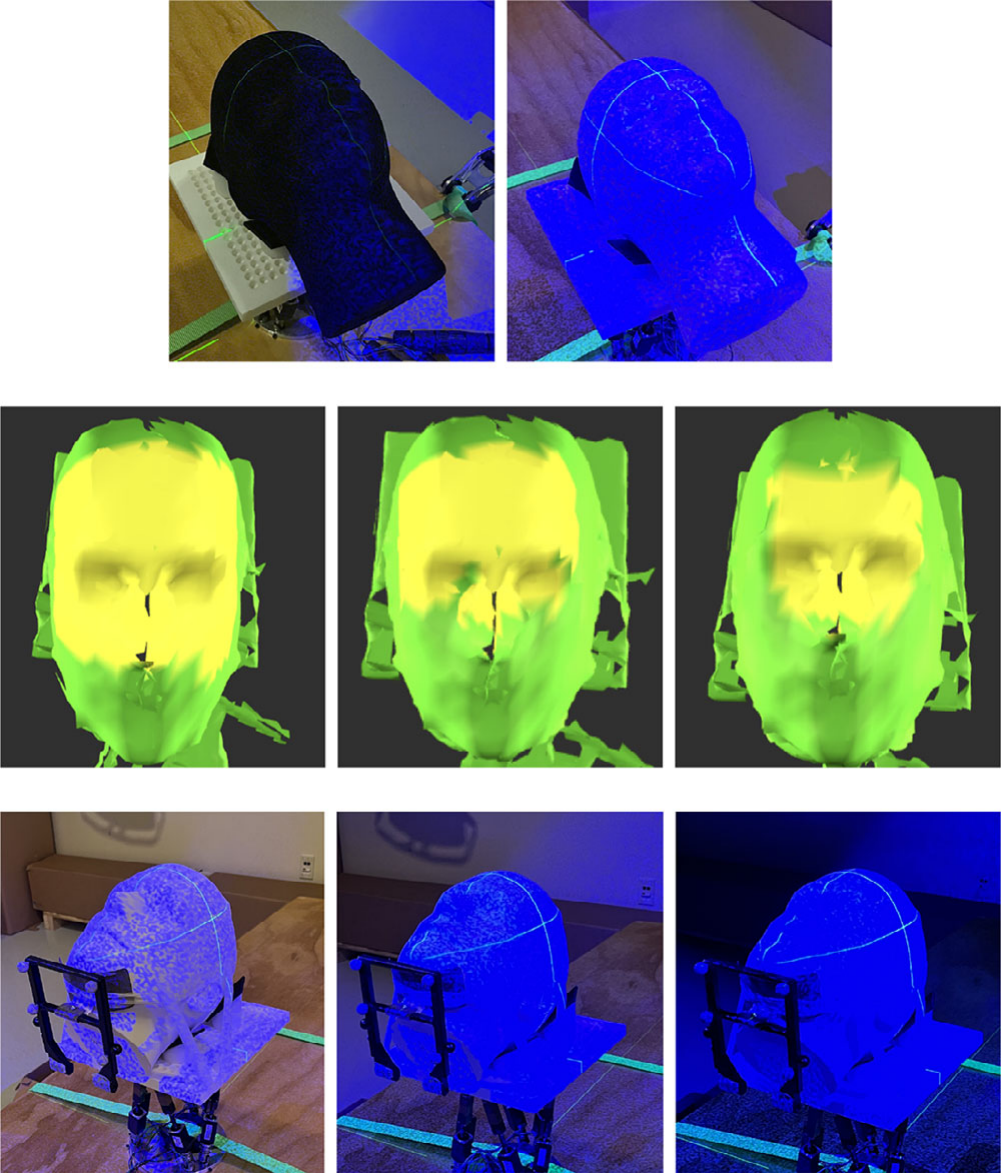
Varying clinical condition tests include surface color, ROI selection and room light conditions. First row: black and white color head phantoms. Second row: large, middle, and small head ROI selections (shown in yellow). Third row: bright, normal, and dark (no light) room lighting conditions. ROI, region of interest.

A variety of clinical conditions involving surface color, treatment room lighting, and ROI selections were considered (Figure 2). These tests align well with the acceptance tests as recommended in the ESTRO‐ACROP report on SGRT devices.^2^ For different skin types two Styrofoam head phantoms were used that were identical except one being black and the other white. For ambient light, three different conditions were taken into account (bright, normal, and dark). The ‘bright’ condition involved turning on all the lights in the room, while the ‘dark’ condition entailed turning off all the lights. The ‘normal’ condition represented an intermediate level of brightness between the ‘bright’ and ‘dark’ conditions.

In general, ROI surface selection plays a crucial role in determining the accuracy and image processing time of surface tracking systems. Here, three ROI sizes were investigated (small, medium, and large). The large ROI corresponded to the maximum possible clinically recommended head tracking ROI, as it encompasses the largest area of stable anatomical features while excluding non‐rigid features such as the jaw or hair.^24^, ^25^ The medium and small ROIs were subsets of the large ROI. The small ROI encompassed the forehead, eyes, eyebrows, and portions of the cheeks and nose. The medium ROI represented an intermediate size, encompassing an area between the small and large ROIs.

### 2.3 Temporal performance evaluation

Two temporal specifications were evaluated in this work: frame rate and measurement time lag. All optical tracking systems require processing time for each measurement, and shorter processing times indicate a faster response of the tracking system. In the evaluation setup, the IR camera and robot system were controlled by the same computer using LabView, whereas, the LUNA 3D system was controlled by its own computer. To allow a common time frame, both of these computers were then synchronized with the online Coordinated Universal Time (UTC) standard. The robot moved the head phantom along predefined trajectories, and both the LUNA 3D and IR tracking systems recorded the dynamic head trajectory. Three times stamps were therefore generated (robot, LUNA 3D, and IR). Similar to the varying clinical conditions experiment, the robot was programmed to execute the same cube motion.

The trajectories in the robot coordinate system for the hexapod robot, IR, and LUNA 3D measurements were denoted as H(t), R(t), and L(t), respectively. Here one should note that R(t) and L(t) were discrete time sequences with different time stamps. The robot's trajectory was shifted backward by a time offset denoted as Δt. For a given time point $t_{i}+\Delta t$, linear interpolation was applied to compute L(t) at this time point $t_{i}+\Delta t$, that is, $L_{t_{i}+\Delta t}$, based on L(t). Subsequently, the difference $R\left(t_{i}+\Delta t\right)-L_{t_{i}+\Delta t}$ was calculated, and the RMSE across all n robot trajectory time stamp points was computed.

To determine the time lag in the LUNA 3D system, the following optimization problem was formulated,

$$Q=\min_{\Delta t}\sqrt{\frac{\sum_{i}^{n}{\left(R\left(t_{i}+\Delta t\right)-P_{t_{i}+\Delta t}\right)}^{2}}{n}}.$$

The optimized value of Δt represents the time lag of the LUNA 3D system, and the corresponding value of Q can be used to assess the difference between the shifted robot trajectory and the LUNA 3D measured trajectory. The time lag of the IR system was also determined using this procedure.

## 3 RESULTS

### 3.1 Translational and rotational accuracy testing

**FIGURE 3:**
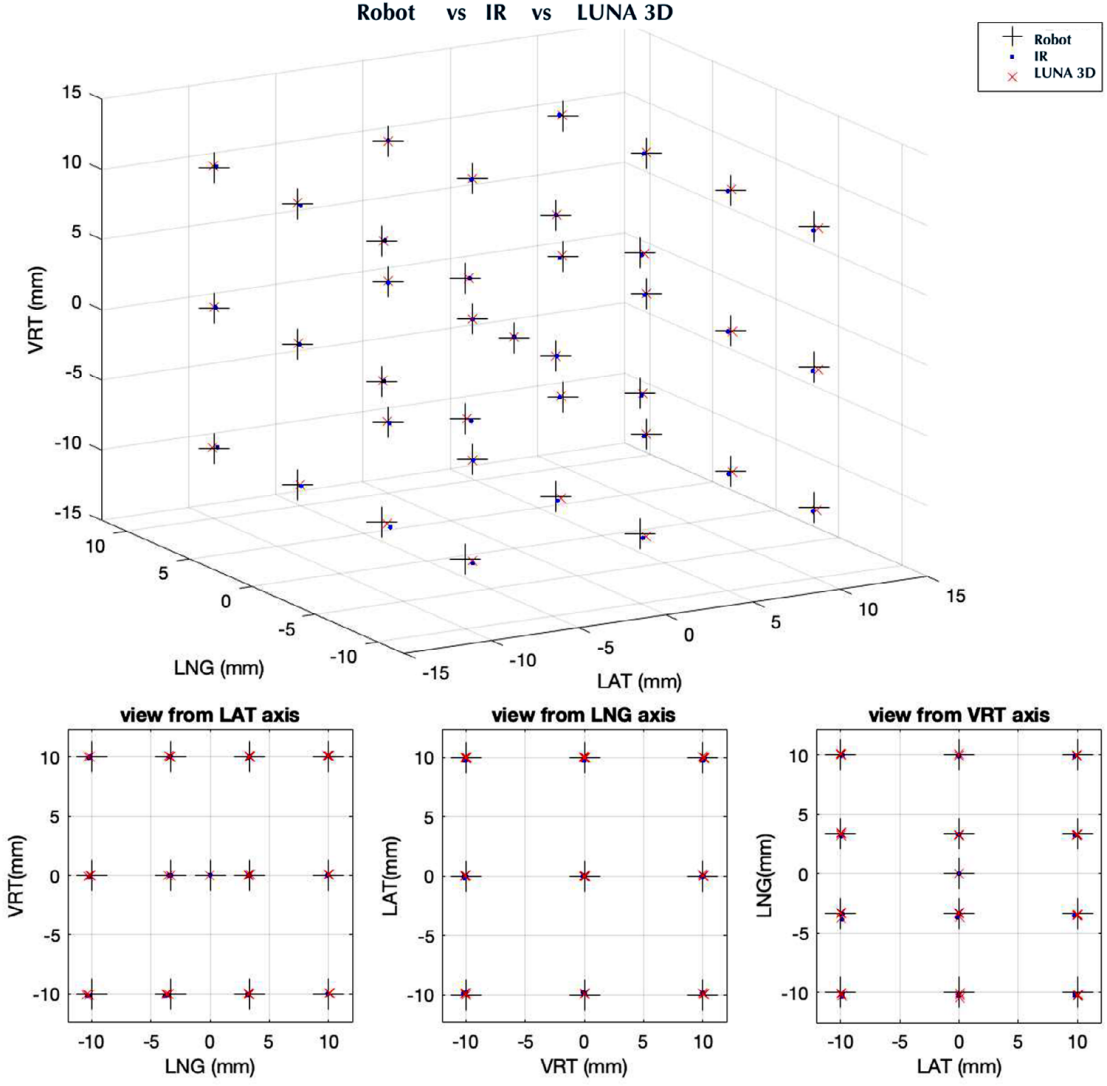
Probed 20 × 20 × 20 ${\mathrm{mm}}^{3}$ volume translational space (left) centered at Linac isocenter. LUNA 3D and IR cameras were compared to 6DoF robot positions. Bottom row shows 2D slices of 3D data taken along each of the six axes. Excellent agreement was shown between robot and measured LUNA 3D and IR positions. 6DoF, 6‐degree‐of‐freedom; Linac, linear accelerator; IR, infrared.

**FIGURE 4:**
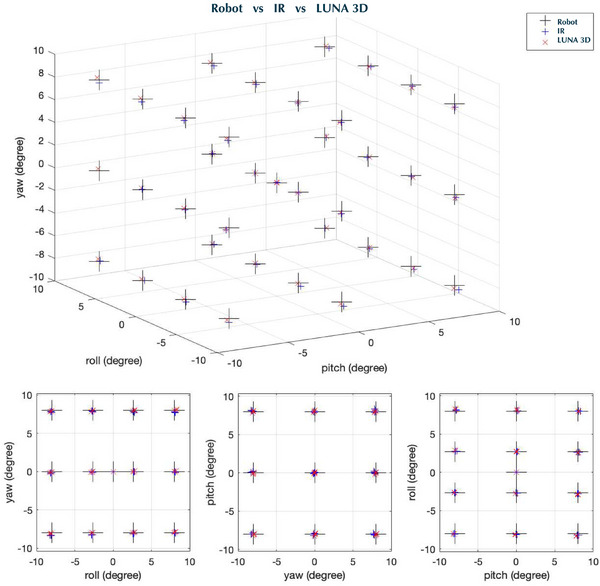
Probed 16 × 16 × 16 ${\text{degree}}^{3}$ volume rotational space centered at Linac isocenter. LUNA 3D and IR cameras were compared to 6DoF robot positions. Bottom row shows 2D slices of 3D data taken along each of the six axes. Excellent agreement was shown between robot and measured LUNA 3D and IR positions. 6DoF, 6‐degree‐of‐freedom; Linac, linear accelerator; IR, infrared.

Figure 3 plots the robot programmed points and corresponding LUNA 3D and IR measured points in transitional space. The same procedure was then repeated for 38 points in a rotational space (Figure 4). To help judge accuracy, 2D slices of the 3D data along LAT, LNG, VRT, pitch, roll, and yaw directions were provided in lower subplots. As can be seen, there was agreement between the robot, LUNA 3D, and IR camera along all dimensions. The median was calculated for each point and then the RMSE was calculated over all 38 points. The RMSE difference between the LUNA 3D and IR over the entire probed volume was found to be extremely small and was approximately 0.01 mm LAT, 0.03 mm LNG, 0.01 mm VRT, 0.02 deg pitch, 0.02 deg roll, and 0.01 deg yaw.

### 3.2 Tracking accuracy under different clinical conditions

The performance of the LUNA 3D under a variety of different clinical conditions that included phantom skin color, treatment room lighting, and different tracking ROI sizes was determined (Figure 2). For each set of conditions, the robot was programmed to move the head phantom to the 8 vertices and the center of a 20 × 20 × 20 ${\mathrm{mm}}^{3}$ cube centered at Linac isocenter (9 points in total). The resultant positional data was then anaylzed and the RMSE calculated between the robot, LUNA 3D, and IR systems. Table 1 summarizes the translational differences among robot position, LUNA 3D and IR measurements for various clinical conditions. In general, it was found that the LUNA 3D was invariant to surface color, room lighting conditions, and different ROI sizes.

**TABLE 1:** LUNA 3D performance under varying clinical conditions. RMSE differences between robot position, IR and LUNA 3D measurements are shown for phantom surface (light and dark), tracking ROI size (small and large), and room lighting (bright and dark).

	Robot ‐ IR			Robot ‐ LUNA 3D			IR ‐ LUNA 3D		
Difference RMSE (mm)	LAT	LNG	VRT	LAT	LNG	VRT	LAT	LNG	VRT
Dark‐large‐bright	0.27	0.02	0.07	0.19	0.05	0.05	0.09	0.05	0.07
Dark‐large‐dark	0.27	0.08	0.07	0.20	0.06	0.05	0.09	0.05	0.08
Dark‐small‐bright	0.24	0.07	0.09	0.16	0.06	0.04	0.10	0.04	0.09
Dark‐small‐dark	0.23	0.08	0.09	0.16	0.10	0.05	0.09	0.07	0.10
Light‐large‐bright	0.25	0.08	0.08	0.17	0.05	0.05	0.09	0.05	0.07
Light‐large‐dark	0.39	0.13	0.08	0.18	0.09	0.06	0.11	0.07	0.06
Light‐small‐bright	0.43	0.36	0.08	0.42	0.34	0.06	0.06	0.12	0.06
Light‐small‐dark	0.45	0.37	0.07	0.42	0.36	0.06	0.06	0.14	0.04

Abbreviations: IR, infrared; LAT, lateral; LNG, longitudinal; RMSE, root‐mean‐square‐error; ROI, region of interest; VRT, vertical.

The camera tracking noise was determined by holding the phantom in a static position at Linac isocenter while repeating the previous variation in skin color, ROI size, and lighting conditions. The results for these tests are summarized in Table 2. Here it was found that there was no observable positional drift over time and that there were only minor noise differences observed across the various conditions. It is also worth noting that the secondary check IR camera also shows very close agreement with the LUNA 3D.

**TABLE 2:** LUNA 3D measurement noise RMSE in vary clinical conditions.

	Translation (mm)			Rotation (degree)		
Noise RMSE (mm)	LAT	LNG	VRT	pitch	roll	yaw
Dark‐large‐bright	0.04	0.05	0.03	0.03	0.04	0.04
Dark‐large‐dark	0.03	0.05	0.03	0.03	0.04	0.04
Dark‐small‐bright	0.07	0.07	0.03	0.04	0.06	0.06
Dark‐small‐dark	0.07	0.06	0.03	0.04	0.05	0.06
Light‐large‐bright	0.02	0.03	0.02	0.02	0.02	0.03
Light‐large‐dark	0.03	0.03	0.02	0.01	0.03	0.02
Light‐small‐bright	0.04	0.03	0.02	0.04	0.03	0.03
Light‐small‐dark	0.04	0.03	0.02	0.02	0.02	0.03

*Note*: Skin: dark and light; ROI size: large and small; Brightness: bright and dark.

Abbreviations: LAT, lateral; LNG, longitudinal; RMSE, root‐mean‐square‐error; ROI, region of interest; VRT, vertical.

### 3.3 Temporal performance

**FIGURE 5:**
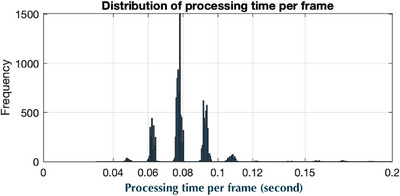
LUNA 3D frame processing time.

**FIGURE 6:**
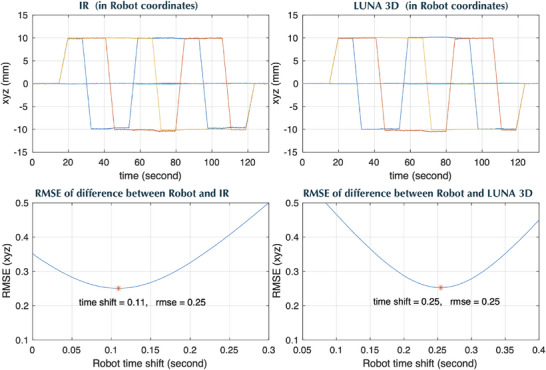
IR and LUNA 3D recorded measurements of a head phantom undergoing a predefined dynamic robot trajectories where blue, yellow and orange lines corresponding to *x*, *y*, and *z* positions, respectively (top row). Temporal delay was calculated be minimizing the RMSE between the robot and camera trajectories as a function of temporal shift. (bottom row). IR, infrared; RMSE, root‐mean‐square‐error.

Temporal performance was determined by tracking the head phantom undergoing a well‐defined dynamic 6D motion trajectory (Figure 6). The temporal difference between adjacent captured frames were calculated and binned to generate the histogram shown in Figure 5. For the LUNA 3D it was found that the frame processing time was 0.080 ± 0.013 s with 98.5% of frames having a processing time of less 0.11 s. For 10 tests involving different ROI sizes, the measured frame rate was found to be 12.29–12.46 frame/second. The temporal lag was calculated to be 250ms, or approximately two frames delay time.

## 4 DISCUSSION

In this work the spatial and temporal performance of the LUNA 3D system was investigated with use of a 6D robot phantom and IR marker tracking for additional position verification. The primary innovation of this work was verification that use of blue light projection together with narrow bandpass filters and advanced triggering can allow robust tracking for different surface colors and ambient room light conditions. This helps resolve a long standing clinical issue as many current SGRT systems have problems accurately rendering the 3D surface over a wide range of skin tones.^1^ Being insensitive to skin and room light variations, the LUNA 3D further simplifies the technicalities of SGRT as it does not require manual adjustment of camera apertures or software based selection of skin type before treatment. As shown in Table 1, for varying surface colors, ambient light, and ROI sizes the max position deviation of the LUNA 3D compared to the robot and IR camera was found to be 0.42 mm and 0.14 mm, respectively.

Measurements from the LUNA 3D exhibited closer agreement with the IR camera than with the 6D robot. This discrepancy was attributed to the LUNA 3D exceeding the mechanical tolerances of the robot which is estimated to be limited to approximately 0.3 mm when moving within a cubic workspace of side length of 20 cm. These limitations in robot accuracy can be attributed to a wide variety of factors such as U‐joint backlash, sub‐millimeter errors in linear actuator lengths, or other mechanical limitations. On the other hand, the IR camera used in this study is well‐known and well‐characterized for its high accuracy.^17^, ^26^ The high agreement between the LUNA 3D and IR camera demonstrates that it is possible for an SGRT system to operate at the accuracy level of IR marker tracking. Such high SGRT accuracies can be useful for high‐precision stereotactic radiosurgery (SRS) or stereotactic body RT (SBRT) treatments as well as newer robotic SRS methods that require continuous sub‐millimeter and sub‐degree head position feedback for motion control.^21^, ^27^, ^28^ Additionally, to further evaluate the performance of the LUNA 3D system in a clinical setting, verification at different isocenters using radiographic imaging would be valuable. However, this is beyond the scope of the current study.

The choice of ROI size typically impacts the accuracy and processing time of SGRT systems.^17^ Smaller ROIs generally lead to faster processing times but may compromise tracking accuracy due to reduced surface feature information. Larger ROIs, on the other hand, provide more feature information, potentially improving tracking accuracy but at the cost of increased processing time. This decreasing frame rate as a function of the increasing ROI area effect was not observed for the LUNA 3D. For the small, medium, and large ROIs used during the study it was the frame rate was approximately constant at 12.5 fps (Figure 5). This was the result of high speed GPU based rendering of 3D surface where the entire observed surfacewas rendered at 12.5 fps regardless of ROI size. These excellent results in terms of ROI accuracy and temporal performance likely make the LUNA 3D system suitable for clinical RT procedures involving precise 6D positioning (SRS, H&N, and SBRT) as well as real‐time adaptive or gated treatments. An other potential future use case could include proton FLASH for head and neck (H&N) RT.^29^ In FLASH, it is critical that the patient maintains the correct setup position until the moment the beam is activated, as rapid delivery (< 1 s) makes intrafractional beam gating challenging or impossible. With its capability to track an ROI at high temporal speeds, the LUNA 3D system could enable real‐time H&N setup position monitoring and facilitate a quick treatment termination if any setup deviations are detected.

Although the 250 ms lag time was found to be lower than other commercial SGRT systems,^30^ it still exceeds the AAPM TG‐142 recommended lag time of ≤ 100 ms for a gating system.^31^ However, it is important to note that the LUNA 3D commercial gating system is still under development and has not yet been released. Given that LUNA 3D maintains an almost constant frame rate of 12.5 fps (80 ms), which does not vary with ROI size, it is likely that a lag time of < 100 ms can be achieved. The reported 250 ms lag time was derived from LUNA 3D log files, which were not optimized for real‐time response and included file data buffering, memory‐to‐disk writing, and other processes contributing to lag time beyond the 80 ms frame rate.

Although our results indicate that the LUNA 3D system demonstrates good static accuracy for patient positioning, several key areas require further investigation. Future studies should explore the impact of camera occlusion on the fidelity of surface capture, as well as incorporate rigorous radiographic isocenter verification to confirm spatial alignment. Additionally, the effects of couch rotations on tracking accuracy, beam‐hold performance during treatment delivery, and systematic assessment of respiratory motion tracking under dynamic conditions need to be explored. While the LUNA 3D system demonstrates high precision under various conditions, it is also important to consider the potential limitations arising from the use of DICOM‐imported reference surfaces. Reference surfaces acquired directly via SGRT offer inherently higher spatial resolution than those imported from DICOM data. In DICOM‐based reconstruction, the “body” or “external” contour is defined by a selected Hounsfield Unit (HU) threshold in the treatment planning system (TPS), a process that can introduce systematic errors–especially in small treatment regions such as the head and neck where complex structures like the nose may be inadequately rendered. Moreover, inconsistencies in TPS protocols, such as the variable inclusion of the immobilization mask, can lead to discrepancies on the order of millimeters. These limitations underscore the clinical advantage of an open mask approach in SGRT, where real‐time, high‐resolution surface capture circumvents the uncertainties associated with HU‐dependent segmentation. Further investigation is needed to quantify these errors and refine patient setup strategies, as highlighted in the ESTRO‐ACROP guidelines.^2^

## 5 CONCLUSIONS

A novel SGRT camera was characterized, showing that the use of blue‐light projection, narrow bandpass filtering, and advanced triggering allows robust tracking across different skin colors and ambient room light conditions. GPU parallel processing allowed rendering of 3D surfaces in real‐time at a constant frame rate regardless of the tracked ROI size on a head phantom.

## CONFLICT OF INTEREST STATEMENT

The authors declare no conflicts of interest.
